# Fluorine-18-Labeled Positron Emission Tomography Probe Targeting Activated p38α: Design, Synthesis, and In Vivo Evaluation in Rodents

**DOI:** 10.3390/ph18040600

**Published:** 2025-04-20

**Authors:** Mikiya Futatsugi, Anna Miyazaki, Yasukazu Kanai, Naoya Kondo, Takashi Temma

**Affiliations:** 1 Department of Biofunctional Analysis, Graduate School of Pharmaceutical Sciences, Osaka Medical and Pharmaceutical University, 4-20-1 Nasahara, Takatsuki 569-1094, Osaka, Japan; ompu72123057@s.ompu.ac.jp (M.F.); anna.miyazaki@ompu.ac.jp (A.M.); kondon@hirakata.kmu.ac.jp (N.K.); 2 Kansai BNCT Medical Center, Educational Foundation of Osaka Medical and Pharmaceutical University, 2-7 Daigakumachi, Takatsuki 569-8686, Osaka, Japan; yasukazu.kanai@ompu.ac.jp; 3 Division of Fundamental Technology Development, Near InfraRed Photo-ImmunoTherapy Research Institute, Kansai Medical University, 2-5-1 Shin-machi, Hirakata 573-1010, Osaka, Japan

**Keywords:** p38α, brown adipose tissue, positron emission tomography, ^18^F fluorination, molecular imaging, diabetes, obesity

## Abstract

**Background/Objectives:** The kinase p38α, a member of the mitogen-activated protein kinase (MAPK) family, is activated by external stimuli and plays a crucial role in inflammation, tumor growth, and metabolic disorders. In particular, p38α is involved in thermogenesis and the metabolism of glucose in brown adipose tissue (BAT), and it contributes to the suppression of obesity and diabetes. The noninvasive imaging of activated p38α could help elucidate diverse pathological processes, including metabolic and inflammatory conditions. This study aimed to develop and evaluate a novel fluorine-18-labeled positron emission tomography (PET) probe for imaging activated p38α in vivo. **Methods:** We designed 6-(4-[^18^F]fluoro-2-fluorophenoxy)-8-methyl-2-(tetrahydro-2*H*-pyran-4-ylamino)-pyrido[2,3-*d*]pyrimidin-7(8*H*)-one ([^18^F]R1487) by replacing a fluorine atom in R1487, which is a highly selective p38α inhibitor, with ^18^F. A tributylstannyl precursor was reacted with [^18^F]KF in the presence of a copper catalyst to synthesize [^18^F]R1487. Biodistribution studies and PET/computed tomography (CT) were performed on normal mice to evaluate the in vivo potential of [^18^F]R1487. **Results:** [^18^F]R1487 was obtained with a decay-corrected radiochemical conversion of 30.6 ± 5.6% and a decay-corrected radiochemical yield of 6.9 ± 3.6% with a radiochemical purity of >99% after reversed-phase high-performance liquid chromatography purification. The biodistribution study demonstrated high and rapid radioactivity accumulation in BAT (16.3 ± 2.7 %ID/g at 5 min post-injection), with a consistently high BAT-to-blood ratio (>5 over 2 h post-injection). PET/CT imaging successfully visualized BAT with high contrast. **Conclusions:** These results suggest that [^18^F]R1487 is a promising PET probe for imaging activated p38α in vivo, which has potential applications for pathophysiological conditions such as inflammation, cancer, and metabolic disorders.

## 1. Introduction

The kinase p38, a member of the MAPK family of serine/threonine kinases, is activated in response to external stimuli [1,2]. Among the isoforms of p38, activated p38α is well-known for its role in releasing inflammatory cytokines. p38α promotes tumor cell growth by enhancing the activity of transcription factors in tumors [3], and it plays a critical role in suppressing obesity and diabetes through its involvement in thermogenesis and the metabolism of glucose in brown adipose tissue (BAT) [4,5]. Given these diverse roles, the noninvasive imaging of activated p38α is expected to provide significant insights into the pathology of various diseases and to aid in their diagnoses.

**Figure 1:**
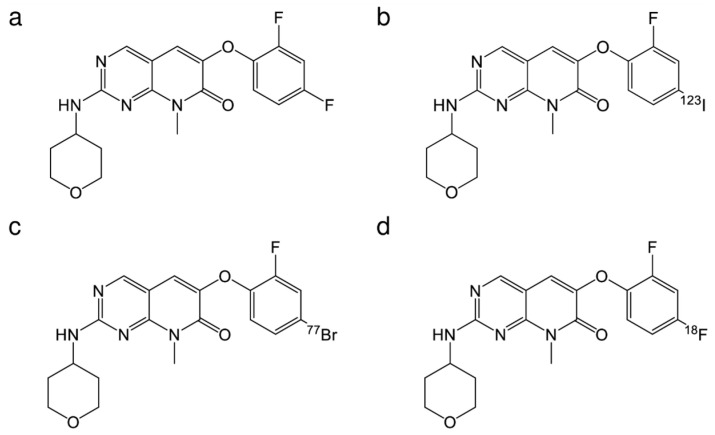
Chemical structures of (**a**) R1487, (**b**) [^123^I]4-IR, (**c**) [^77^Br]4-BR, and (**d**) [^18^F]R1487.

R1487 (Figure 1a), which is a highly selective p38α inhibitor, has been reported to suppress the production of TNF-α and IL-6 following oral administration [6]. In monkeys, its oral bioavailability and half-life in blood were 51.6% and 22.5 h, respectively, indicating excellent membrane permeability and stability in vivo [6]. According to this, we anticipated that it would have potential to serve as a lead compound for intravenously administrable in vivo imaging probes. Thus, we previously designed 6-(4-[^123^I]iodo-2-fluorophenoxy)-8-methyl-2-(tetrahydro-2*H*-pyran-4-ylamino)-pyrido[2,3-*d*]pyrimidin-7(8*H*)-one ([^123^I]4-IR) and 6-(4-[^77^Br]bromo-2-fluorophenoxy)-8-methyl-2-(tetrahydro-2*H*-pyran-4-ylamino)-pyrido[2,3-*d*]pyrimidin-7(8*H*)-one ([^77^Br]4-BR) based on R1487 (Figure 1b,c), and we demonstrated their effectiveness as in vivo imaging probes for activated p38α with single photon emission computed tomography (SPECT) [7,8]. Notably, [^125^I]4-IR exhibited high in vivo stability and the preferential accumulation of radioactivity proportional to the activated p38α expression. However, the p38α inhibitory activities of these compounds were slightly lower than those of the lead compound. This reduction is likely due to the introduction of a larger halogen atom, such as iodine or bromine, at the para position of the phenoxy group, which replaces the smaller fluorine originally present in R1487. The phenoxy moiety is recognized as a key site for interaction between p38α and R1487 [6].

In this study, we designed 6-(4-[^18^F]fluoro-2-fluorophenoxy)-8-methyl-2-(tetrahydro-2*H*-pyran-4-ylamino)-pyrido[2,3-*d*]pyrimidin-7(8*H*)-one ([^18^F]R1487) by substituting fluorine at the para position of the R1487 structure with ^18^F, which is a positron-emitting radiofluorine with a physical half-life of 109.8 min (Figure 1d). This isotopic radiolabeling strategy was expected to retain the p38α inhibitory activity of R1487, surpassing the inhibitory effects of [^123^I]4-IR and [^77^Br]4-BR while enabling positron emission tomography (PET) imaging with high in vivo quantification capability. Here, we successfully synthesized [^18^F]R1487 with high radiochemical purity (RCP) and evaluated, using PET, its potential as an in vivo imaging probe for activated p38α in the BAT of normal ddY mice, a commonly used outbred strain for pharmacological and imaging studies.

## 2. Results

### 2.1. Radiosynthesis

**Scheme 1:**
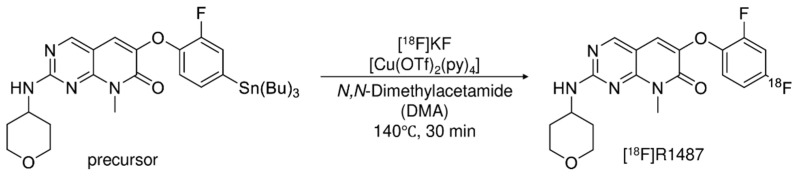
Radiosynthesis scheme for [^18^F]R1487.

**Figure 2:**
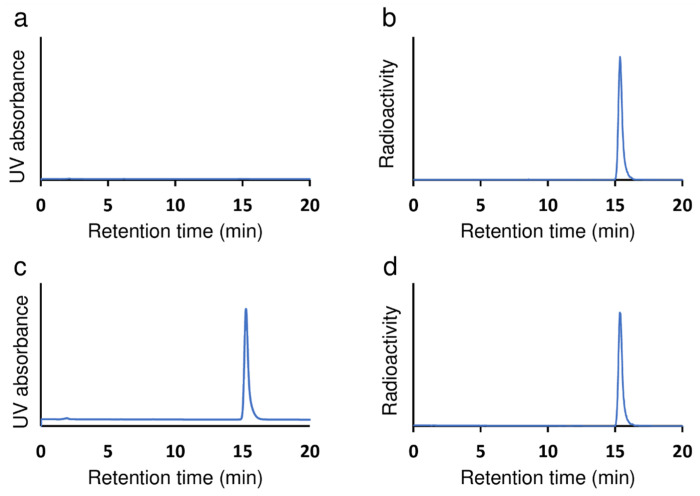
Analytical HPLC chromatograms of [^18^F]R1487. (**a**) UV chromatogram of the final solution containing [^18^F]R1487 under carrier-free conditions. No prominent UV peak is observed. (**b**) Corresponding RI chromatogram of the same sample, showing a clear radioactive peak at 15.7 min. (**c**) UV chromatogram of [^18^F]R1487 solution spiked with cold R1487 (carrier-added), clearly displaying a peak at 15.7 min. (**d**) Corresponding RI chromatogram of the carrier-added sample, showing a clear radioactive peak at 15.7 min. All chromatograms were recorded under identical analytical conditions, and the UV chromatograms (**a**,**c**) were plotted using the same vertical axis scale to enable direct comparison of signal intensity. The coincidence of radioactivity and UV peaks confirms the chemical identity. Conditions for RP-HPLC: column, COSMOSIL 5C18-AR II (10 I.D. × 250 mm); flow rate, 5 mL/min; mobile phase, water/acetonitrile = 60:40 (isocratic).

[^18^F]R1487 was synthesized from its corresponding tributylstannyl precursor, as is shown in Scheme 1. Details of the synthesis procedure, including chemical materials used, reaction conditions, and purification steps, are available in our previously published work [7]. The decay-corrected radiochemical conversion (RCC) of the radiofluorination reaction was 30.6 ± 5.6%, whereas the decay-corrected radiochemical yield (RCY) was 6.9 ± 3.6% (*n* = 3). Following purification by reversed-phase high-performance liquid chromatography (RP-HPLC), [^18^F]R1487 was obtained with an RCP exceeding 99% (*n* = 3), as shown in Figure 2. The total synthesis time from the initiation of [^18^F]HF was 126.7 ± 16.2 min (*n* = 3). The molar activity was estimated to be 1983 GBq/μmol.

### 2.2. In Vitro Assay

The log *D* value of [^18^F]R1487 was determined to be 2.2, which suggests that it had moderate to high hydrophobicity. After a 2 h incubation of [^18^F]R1487 in mouse plasma, more than 96% of the radioactivity was detected in its intact form by normal-phase thin-layer chromatography (TLC) analysis. This result confirmed the high in vitro stability of [^18^F]R1487 and suggests that it would be suitable as a radiotracer for in vivo applications.

### 2.3. Biodistribution Study

Following the intravenous administration of [^18^F]R1487, radioactivity was rapidly distributed across various tissues, with particularly high accumulation observed in BAT at 5 min post-administration (16.3 ± 2.7 %ID/g), as shown in Table 1. The radioactivity in BAT decreased to approximately half of its initial level at 15 min (8.0 ± 2.6 %ID/g) and dropped further, to levels that were comparable to those in other tissues at 60 min post-administration. The BAT/muscle radioactivity ratio was high at 5 min (6.1 ± 1.4), and it decreased by half to 3.3 ± 0.3 at 15 min and continued to decline gradually thereafter. In contrast, the BAT/blood radioactivity ratio increased between 5 and 15 min post-administration (5 min: 5.8 ± 1.0; 15 min: 8.1 ± 1.7), which suggests that the active delivery of radioactivity to BAT occurred during the early phase and remained above 5 for up to 120 min. Radioactivity initially accumulated in the liver (11.3 ± 1.9 %ID/g) and shifted over time to the small and large intestines, suggesting a hepatobiliary excretion pathway.

### 2.4. In Vivo Inhibition Study

**Figure 3:**
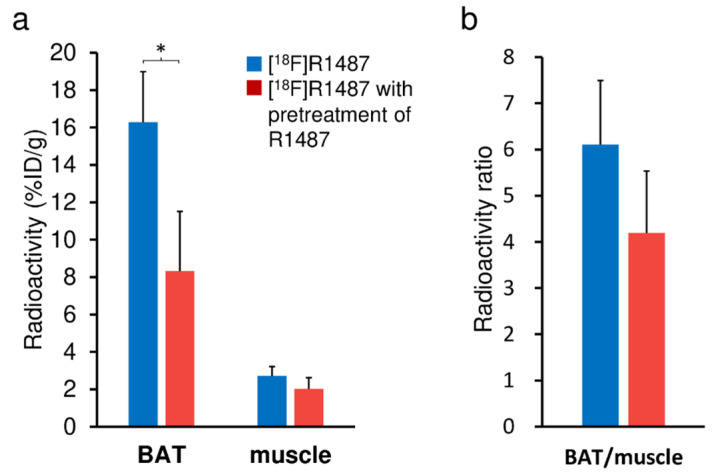
In vivo inhibition of [^18^F]R1487. (**a**) Radioactivity accumulation in BAT and muscle. (**b**) Radioactivity ratio of BAT and muscle. Data represent mean %ID ± standard deviations (SD), *n* = 4 or 5 * *p* < 0.05.

An in vivo blocking study was performed, in which mice were intraperitoneally pretreated with 30 mg/kg of nonradioactive R1487 60 min prior to intravenous [^18^F]R1487 administration. As shown in Figure 3, BAT radioactivity was significantly reduced and the BAT-to-muscle ratio also showed a decreasing trend, supporting in vivo specificity of [^18^F]R1487.

### 2.5. PET/CT Imaging

**Figure 4:**
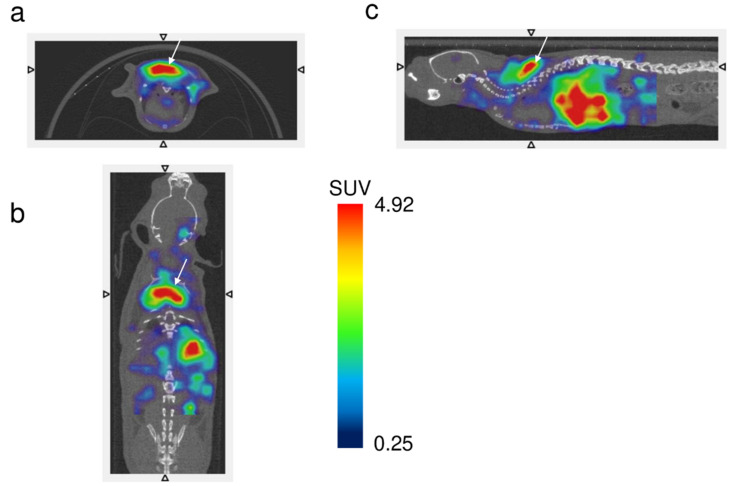
Representative PET/CT images of a normal mouse, obtained with [^18^F]R1487 (3.0 MBq). PET and CT scans were performed sequentially. (**a**) Transverse, (**b**) coronal, and (**c**) sagittal PET images acquired 10–15 min post-administration are displayed as fused PET/CT images. White arrow indicates BAT. Notable abdominal accumulation observed is consistent with hepatobiliary excretion of [^18^F]R1487.

Figure 4 presents representative PET images fused with computed tomography (CT) images, obtained 10–15 min post-intravenous administration of [^18^F]R1487 in a normal mouse. As indicated by the white arrows, BAT was clearly visualized in the images. The mean standardized uptake value (SUV) in the BAT, calculated by manually defining a region of interest (ROI) over the BAT, was 4.7, which indicates substantial tracer uptake in the BAT. The radioactivity in the excised BAT was immediately measured after PET/CT imaging using a γ counter and determined to be 11.6 %ID/g.

## 3. Discussion

p38α is activated in the early stages of inflammatory diseases, in which it plays a key role in the release of inflammatory cytokines [1,9]. In tumors, p38α has a dual function [10,11,12]: it acts as a suppressor before tumor formation, but it promotes tumor growth once a tumor has developed. p38α is also involved in blood glucose regulation through thermogenesis and glucose metabolism in BAT [13], thus BAT is considered to be a potential therapeutic and diagnostic target for metabolic diseases, particularly diabetes [4,5]. Considering this, we selected BAT as the primary tissue with which to evaluate the potential of a novel imaging probe that targets activated p38α.

R1487 is a p38α-selective inhibitor with high potency and specificity. Its selectivity for p38α over other p38 isoforms was reported with a Kd value of 0.2 nM, and it showed at least 145-fold selectivity over p38β, γ, and δ [6]. Pharmacokinetic studies have been conducted to evaluate the absorption, distribution, metabolism, and excretion properties of R1487, which shows a bioavailability of 51.6% following oral administration in monkeys, a half-life of 22.5 h, and predominantly hepatic clearance [6]. These properties indicate that radiolabeled R1487 would achieve systemic exposure levels that are suitable for imaging or therapeutic applications via intravenous administration. Regarding safety, previous preclinical studies have assessed the toxicity profile of R1487. Acute and chronic toxicity tests in rats demonstrated acceptable tolerability up to 10 mg/kg, with no severe adverse events observed [6]. This tolerability suggests a reasonable safety margin for in vivo applications, especially for molecular imaging probes that are generally used in trace amounts in vivo. Based on these favorable properties of R1487, we aimed to develop an in vivo molecular imaging probe that selectively targets activated p38α under various pathophysiological conditions and a specifically designed [^18^F]R1487, in which the fluorine in the structure of R1487 is replaced with ^18^F, a positron emitter, for application in PET. In fact, [^18^F]R1487 demonstrated a log *D* value of 2.2, suggesting adequate lipophilicity for efficiently targeting an intracellular biomolecule like activated p38α and further supporting favorable physicochemical properties for its in vivo use [14].

We successfully synthesized [^18^F]R1487 using a tributylstannyl precursor for radiolabeling. This precursor was selected because we had previously established a synthetic method for obtaining [^123/125^I]4-IR [7] and [^77^Br]4-BR [8] via an organotin–radiohalogen exchange reaction, which facilitated its application in this study. Notably, the RCC and RCY of [^18^F]R1487 obtained with this method were sufficient for conducting stability analysis, a biodistribution study, and PET/CT imaging, demonstrating the practical feasibility of [^18^F]R1487 for molecular imaging applications. Although the RCY was low (<7%) relative to the RCC (~30%), optimizing the HPLC separation method could potentially improve the RCY. In addition, previous studies have suggested that using a pinacolborane ester precursor could enhance the efficiency of the radiofluorination reaction relative to using a tributylstannyl precursor [15,16]. A direct comparison between tributylstannyl and pinacolborane ester precursors, as well as the optimization of the radiolabeling conditions—including the choice of copper catalyst and reaction solvent, automated radiosynthesis procedures, and purification processes—would increase the availability of [^18^F]R1487 by potentially improving labeling efficiency. This could facilitate broader applications in PET imaging in the future, including those requiring high-activity synthesis for preclinical or clinical use.

The study of the biodistribution of [^18^F]R1487 demonstrated the highest radioactivity at 5 min post-injection in BAT among all organs analyzed. This early and prominent accumulation suggests a strong initial delivery to BAT, reinforcing its potential as a molecular imaging probe for activated p38α-expressing tissues. The in vivo inhibition experiment further substantiates the specificity of [^18^F]R1487 for activated p38α, as pretreatment of cold R1487 markedly reduced BAT radioactivity. This finding aligns with the proposed mechanism of selective p38α targeting. Notably, this uptake pattern differed from those observed for the structurally related probes [^12^⁵I]4-IR [7] and [⁷⁷Br]4-BR [8], which reached peak radioactivity accumulation at 30 min and 15 min post-injection, respectively, in inflamed tissues, which were used as target sites for activated p38α-expressing tissues. Given that the p38α binding affinity follows the order R1487 > 4-BR > 4-IR, this observation implies a correlation between the binding affinity and the rate of accumulation in the target tissues. However, further studies are needed to confirm this relationship and evaluate its implications for the design of probes. Although the radioactivity in BAT declined after its peak at 5 min post-injection, the BAT-to-blood ratio increased from 5.8 at 5 min to 8.1 at 15 min post-injection, suggesting that radioactivity was not simply delivered passively into BAT but actively targeted BAT where activated p38α was highly expressed. Moreover, [^18^F]R1487 maintained a relatively high BAT-to-blood ratio (above 5) for up to 2 h post-injection. Specifically, the inflamed site-to-blood ratios at 2 h were 4.1 and 4.4 for [^77^Br]4-BR and [^125^I]4-IR, respectively, whereas [^18^F]R1487 demonstrated a slightly higher ratio of 5.9. Although BAT and inflamed tissues have different functions, their shared characteristic of elevated p38α activation suggests that [^18^F]R1487 may exhibit kinetics comparable to that of BAT when evaluated in an inflammatory model. Other than in BAT, notable accumulation was observed in the liver at 5 min post-injection, which is consistent with the hepatobiliary clearance pattern previously reported for R1487 [6]. Over time, increasing radioactivity levels were detected in the small and large intestines, further supporting that [^18^F]R1487 follows the same hepatic excretion pathway. Additionally, some degree of radioactivity accumulation was observed in the bone. However, no trend of rapid increase was detected during the experimental time window, and [^18^F]R1487 was highly stable in an in vitro stability assay, suggesting that this bone accumulation is unlikely to have resulted from the in vivo defluorination of [^18^F]R1487. This interpretation is further supported by previous reports showing in vivo defluorination [17,18]. In vivo stability assay was not performed in this study, but the high in vivo stability of R1487 was previously reported in rats and monkeys [6]. The strong in vitro stability observed in this study, together with the reported in vivo stability of R1487, supports the assumption that [^18^F]R1487 is metabolically stable in vivo. Given that p38α activity has been reported in bone [19,20], further investigation is warranted to elucidate the mechanism underlying the radioactivity accumulation in bone and its potential implications for p38α-targeted imaging.

PET imaging clearly visualized BAT as the [^18^F]R1487 accumulated in the area along the spine near the scapula, where BAT is known to be located [21,22]. This confirmed the potential of [^18^F]R1487 as a molecular imaging probe for activated p38α-expressing BAT. In addition, notable radioactivity accumulation was observed in the abdominal region, which is consistent with the results of the biodistribution study. Ideally, PET imaging should have been initiated simultaneously with the intravenous administration of [^18^F]R1487 to capture its dynamic delivery and distribution from the earliest time points. However, due to technical limitations, imaging was initiated at 5 min post-injection. Despite this, the acquired images successfully demonstrated the early accumulation of radioactivity in the BAT, thereby validating the feasibility of [^18^F]R1487 for activated p38α imaging. Given the anatomical proximity of BAT to the heart, a potential future study would be to refine the field of view and narrow it to extract time–activity curves for BAT and blood in the heart from dynamic PET images. This could provide further quantitative insights into BAT kinetics and activated p38α function in BAT under broad pathophysiological conditions. Additionally, imaging could be performed within a short period (10–15 min) and provide high-contrast PET images. This capability would not only enhance clinical feasibility but would also minimize radiation exposure, making [^18^F]R1487 a promising and safe PET agent for activated p38α imaging.

In this study, we used BAT as a representative tissue with high p38α expression [23] to evaluate [^18^F]R1487. Because BAT is recognized as a suitable physiological model for assessing molecular imaging probes for activated p38α, disease model mice were not employed. This approach allowed us to focus on the fundamental pharmacokinetic properties of [^18^F]R1487 under physiological conditions. Beyond the technical advantages of BAT, which enabled the use of normal mice, its evaluation as a target tissue holds biological significance. Given the critical role of p38α in thermogenesis and glucose metabolism, BAT has been implicated in metabolic diseases such as obesity and diabetes [4,5,13]. Therefore, the noninvasive imaging of activated p38α in BAT using [^18^F]R1487 may contribute not only to the validation of this probe but also to a deeper understanding of BAT function in metabolic regulation. This could provide new insights into the role of BAT in metabolic disorders and its potential as a therapeutic target. Furthermore, disease models such as cancer or inflammation models could provide additional insights into the role of activated p38α in specific pathological conditions [2,3,4,5,13,24,25,26,27]. A comparison of the [^18^F]R1487 uptake with activated p38α expression in such models could help elucidate disease-specific p38α dynamics and further explore the potential applications of [^18^F]R1487 in the diagnosis and therapeutic monitoring of p38α-related diseases. Due to technical constraints associated with limited radioactivity availability and the short half-life of fluorine-18, autoradiographic analysis was not conducted in this study. However, future studies will benefit from such analysis to better visualize the tissue-level distribution of [^18^F]R1487, particularly in disease models.

## 4. Materials and Methods

### 4.1. Reagents and Instruments

We obtained all chemicals from Wako Pure Chemical Industry (Osaka, Japan), Sigma-Aldrich Japan (Tokyo, Japan), Nacalai Tesque (Kyoto, Japan), and Towa Pharmaceutical Co., Ltd. (Osaka, Japan), or Tokyo Chemical Industry (Tokyo, Japan), and we used them without further purification. ^1^H-NMR spectra were measured using a Varian NMR system (^1^H 400 MHz, Agilent Technologies, Santa Clara, CA, USA). The HPLC system consisted of an LC-20AT pump (Shimadzu, Kyoto, Japan), an SPD-20A UV detector (Shimadzu), and a 170 radioisotope detector (Beckman Colter, Brea, CA, USA). RP-HPLC was performed using a COSMOSIL 5C18-AR-II column (10 ID × 250 mm; Nacalai Tesque, Kyoto, Japan). Normal-phase TLC was performed using silica gel TLC plates (TLC silica gel 60F_254_, aluminum sheet 20 × 20 cm, Merck, Darmstadt, Germany). Radioactivity was measured using a γ counter (2480 Wizard^2^, PerkinElmer Japan, Kanagawa, Japan).

### 4.2. Radiosynthesis (Scheme 1)

R1487 and a tributylstannyl precursor for radiofluorination were synthesized following our previously reported methods [7]. [^18^F]HF was produced using an in-house 20 MeV cyclotron (CYPRIS HM-20, Sumitomo Heavy Industry, Ltd., Tokyo, Japan) via the ^18^O(p, n)^18^F nuclear reaction of ^18^O-enriched water and a proton beam. The beam current was set to 20 μA, and the irradiation time was approximately 5 min. [^18^F]HF was trapped on a Sep-Pak Accell Plus QMA Plus Light cartridge (WAT023525, Waters, Milford, MA, USA) and eluted with an aqueous solution containing KOTf/K_2_CO_3_ (125:1, 550 μL). The solution was evaporated and azeotropically dried three times with dry CH_3_CN (1 mL). Its residue was dissolved in dry *N*,*N*-dimethylacetamide (600 μL) and transferred to a conical vial containing the precursor (2.0 mg, 3.0 mmol) and tetrakis(pyridine)copper(II) triflate ([Cu(OTf)_2_(py)_4_], 12.2 mg, 18.0 mmol). The reaction mixture was heated at 140 °C for 30 min. After cooling, the reaction was quenched by adding water (6 mL), and the mixture was passed through a Sep-Pak Plus Light C18 cartridge. The cartridge was washed with water (2 mL) and then eluted with CH_3_CN (2 mL). Ascorbic acid (50 μL, 500 mg/2 mL) was added to the eluate, which was then evaporated to concentrate the solution to approximately 500 μL. The resulting solution was purified by RP-HPLC. The purity of the product was subsequently analyzed using RP-HPLC under the same conditions, with a mobile phase of CH_3_CN/water (60:40) at a flow rate of 5 mL/min. RCC was calculated as the ratio of [^18^F]R1487 radioactivity to the initial radioactivity used for the labeling reaction, based on radio-TLC analysis of the crude reaction mixture immediately after completion and cooling of the reaction. This reflects the efficiency of the radiofluorination step, independent of any purification. RCY was calculated as the ratio of decay-corrected radioactivity of the purified and formulated [^18^F]R1487 to the radioactivity eluted from the QMA cartridge, based on measurements obtained using a γ counter. This reflects the overall efficiency of the radiosynthesis, including labeling, purification, and formulation steps.

### 4.3. Log D Measurement

[^18^F]R1487 was first dissolved in 20 μL of PBS (pH = 7.4), and then 500 μL of 1-octanol and 480 μL of PBS were added to achieve a 1:1 (*v*/*v*) ratio. The mixture was shaken for 10 min at room temperature, followed by centrifugation at 10,000 rpm for 10 min. The radioactivity in each phase was measured using a γ counter. The Log D value was calculated based on the ratio of the concentration of the two phases (*n* = 4).

### 4.4. In Vitro Stability Analysis

All the animal experiments were conducted according to the institutional guidelines for animal experiments, and the study protocol was approved by the Institutional Experimental Animal Committee (Permission Number: AP23-004). Male ddY mice (*n* = 29 in total, 5 weeks old, 21–24 g) were purchased from Japan SLC, Inc. (Hamamatsu, Japan) and housed under a 12 h-12 h/light-dark cycle with free access to food and water for at least 1 week before the experiment. Blood was collected from some of the mice (*n* = 5) following euthanasia under isoflurane anesthesia. [^18^F]R1487 (20 μL, 158 kBq) was incubated with mouse plasma (180 μL) at 37 °C for 5, 30, and 120 min. To precipitate the proteins, CH_3_CN (450 μL) was added, and the mixture was filtered using a Cosmonice W filter (Nacalai Tesque). The resulting filtrate was analyzed by normal-phase TLC with a developing solvent of ethylacetate/hexane (5:1). The TLC plate was divided into sections that were 4 mm in length, and the radioactivity of each segment was measured using a γ counter. The intact rate was calculated as the ratio of the radioactivity in the fraction of interest (Rf = 0.6) to the total radioactivity across all sections.

### 4.5. Biodistribution Study

Mice (*n* = 20, 5 weeks old, 23–29 g) were randomly assigned to five groups (*n* = 4 each). [^18^F]R1487 (482–493 kBq/100 μL saline containing 0.2% Tween 80) was intravenously injected into the mice. The animals were euthanized under isoflurane anesthesia at 5, 15, 30, 60, and 120 min post-injection (*n* = 4 for each time point). Sample size was limited to four animals per group in adherence to ethical guidelines for the reduction of animal use. Target tissues, including BAT, blood, muscle, bone, brain, heart, lung, stomach, pancreas, spleen, kidneys, liver, and small and large intestines, were excised and weighed, and the radioactivity of each tissue was measured using a γ-counter. The accumulation of radioactivity was expressed as the percentage of the injected dose per gram of tissue (%ID/g) for all tissues except for the stomach, for which it was expressed as the percentage of the injected dose per organ (%ID). All data were retained for analysis, as none exceeded the predefined exclusion threshold of ±2 SD. To ensure unbiased results, the experiments were conducted under blinded conditions, with separate investigators for the drug administration and the radioactivity measurements.

### 4.6. In Vivo Inhibition Study

Sixty minutes after R1487 (30 mg/kg) was administered intraperitoneally into the mice (*n* = 6, 5 weeks old, 27–29 g), [^18^F]R1487 (482–493 kBq/100 μL, saline containing 0.2% Tween 80) was injected intravenously. The animals were euthanized under isoflurane anesthesia at 5 min post-injection of [^18^F]R1487, BAT and muscle were excised and weighed, and radioactivity was measured using a γ counter. The accumulation of radioactivity was expressed as %ID/g. Based on an exclusion threshold of ± 2 SD from the mean, one data point was removed for subsequent statistical analysis. To ensure unbiased results, the experiments were conducted under blinded conditions, with separate investigators for the drug administration and the radioactivity measurements.

### 4.7. PET/CT Imaging

Normal ddY mice (*n* = 3, 5 weeks old, 27.9 ± 7.1 g) were intraperitoneally anesthetized with a mixture of three anesthetics: medetomidine (0.3 mg/kg), midazolam (4 mg/kg), and butorphanol (5 mg/kg), which were prepared in saline and administered at 5 mL/kg body weight. [^18^F]R1487 (2.3–3.7 MBq/200 μL saline containing 0.2% Tween 80) was intravenously injected into the tail vein. A 25 min PET scan was initiated at 5 min post-injection, and it consisted of 5 frames with a 5 min acquisition each. A small-animal PET/SPECT/CT scanner (E-Class VECTor^6^CT^SD^, MILabs, Utrecht, The Netherlands) was used, and it was equipped with a high-efficiency general purpose rat and mouse collimator. After the PET imaging, CT imaging was performed (exposure time: 40 ms, tube voltage: 55 kv, tube current: 0.33 mA). After the CT imaging, the mice were immediately euthanized under anesthesia, their BAT was removed and weighed, and its radioactivity was measured (*n* = 1). The raw data obtained from the PET scan were reconstructed using the SROSEM method and superimposed onto the CT images. The ROIs were manually placed on the PET image at sites corresponding to the BAT and the right lower abdomen served as a negative control region. The SUVs were then calculated using Amide software 1.0.6 (Slashdot Media, San Francisco, CA, USA).

### 4.8. Statistics

All data are expressed as means ± SD. Statistical analyses were conducted using unpaired *t*-test via GraphPad Prism 8 (GraphPad Software, Boston, MA, USA). Differences were considered statistically significant at the 95% confidence level (*p* < 0.05).

## 5. Conclusions

We successfully synthesized and evaluated [^18^F]R1487 as a novel PET probe for imaging activated p38α. The probe demonstrated high selectivity and stability, favorable pharmacokinetics, and efficient in vivo accumulation in BAT, which is a tissue that represents high p38α expression. PET imaging confirmed the ability of [^18^F]R1487 to visualize BAT with high contrast, whereas biodistribution studies revealed a rapid and dynamic accumulation pattern. These findings highlight the potential of [^18^F]R1487 not only for imaging BAT function but also in broader applications for investigating p38α-related pathophysiological processes. Further studies incorporating disease models would be valuable in exploring its diagnostic potential in inflammation, cancer, and metabolic disorders.

## Figures and Tables

**Table 1:** Radioactivity biodistribution after intravenous administration of [^18^F]R1487 in normal ddY mice.

	Time After Administration (min)				
	5	15	30	60	120
BAT	16.3 ± 2.7	8.0 ± 2.6	5.2 ± 1.1	2.2 ± 0.6	1.0 ± 0.2
Blood	2.8 ± 0.1	1.0 ± 0.2	0.7 ± 0.1	0.4 ± 0.1	0.2 ± 0.0
Muscle	2.7 ± 0.5	2.4 ± 0.3	2.1 ± 0.2	1.0 ± 0.1	0.5 ± 0.0
Bone	6.4 ± 0.7	5.1 ± 0.9	6.7 ± 1.0	6.9 ± 2.9	8.7 ± 2.3
Brain	2.9 ± 0.5	1.3 ± 0.6	1.0 ± 0.6	0.4 ± 0.2	0.2 ± 0.1
Heart	5.0 ± 0.8	2.8 ± 0.5	2.3 ± 0.7	1.4 ± 0.1	0.7 ± 0.1
Lung	4.0 ± 0.6	3.2 ± 0.8	2.3 ± 0.5	1.3 ±0.2	0.4 ± 0.1
Stomach *	1.1 ± 0.2	1.3 ± 0.5	1.8 ± 0.4	4.1 ± 2.2	1.2 ± 0.5
Pancreas	4.4 ± 0.9	2.4 ± 0.6	1.9 ± 0.1	1.0 ± 0.1	1.0 ± 0.4
Spleen	4.0 ± 0.9	4.5 ± 0.8	3.3 ± 0.5	1.7 ± 0.4	0.9 ± 0.1
Kidney	7.7 ± 1.9	5.1 ± 1.3	3.5 ± 0.6	2.4 ± 0.7	0.7 ± 0.1
Liver	11.3 ± 1.9	5.9 ± 1.3	4.7 ± 0.9	3.4 ± 0.3	1.8 ± 0.9
Small intestine	5.8 ± 1.0	8.6 ± 1.9	16.9 ± 4.0	23.8 ± 3.7	13.0 ± 3.4
Large intestine	2.2 ± 0.7	2.3 ± 0.4	4.3 ± 3.7	4.3 ± 1.6	32.6 ± 8.7
BAT/Blood ratio	5.8 ± 1.0	8.1 ± 1.7	7.9 ± 2.5	5.4 ± 0.9	5.9 ± 1.2
BAT/Muscle ratio	6.1 ± 1.4	3.3 ± 0.3	2.6 ± 0.5	2.3 ± 1.1	1.9 ± 0.5

Data are presented as %ID/g tissue. Each value represents the mean ± SD for four animals at each time point. * represented as %ID.

## Data Availability

All data generated or analyzed in this study are included in this publication.
